# 3,3-Dichloro-1-(chloro­meth­yl)indolin-2-one

**DOI:** 10.1107/S1600536810040717

**Published:** 2010-10-20

**Authors:** Yao Wang, Chong-Qing Wan, Tingting Zheng, Sheng-Li Cao

**Affiliations:** a Department of Chemistry, Capital Normal University, Beijing 100048, People’s Republic of China

## Abstract

In the title compound, C_9_H_6_Cl_3_NO, the pyrrole ring is almost coplanar with the benzene ring [dihedral angle = 1.90 (9)°], while the Cl—C—N—C torsion angle is 98.78 (17)°. In the crystal, pairs of mol­ecules are inter­connected by pairs of Cl⋯Cl inter­actions [3.564 (5) Å], forming dimers, which are further peripherally connected through inter­molecular C—H⋯O=C and π–π inter­actions [centroid–centroid distances = 4.134 (7), 4.134 (6) and 4.238 (7) Å], forming a two-dimensional network.

## Related literature

For the synthesis of the title compound, see: Höhme & Schwartz, (1974 ▶). For the synthesis of 1-(chloro­meth­yl) indoline-2,3-dione, see: Höhme & Schwartz, (1973 ▶). For Cl⋯Cl inter­actions, see: Reddy *et al.* (2006 ▶).
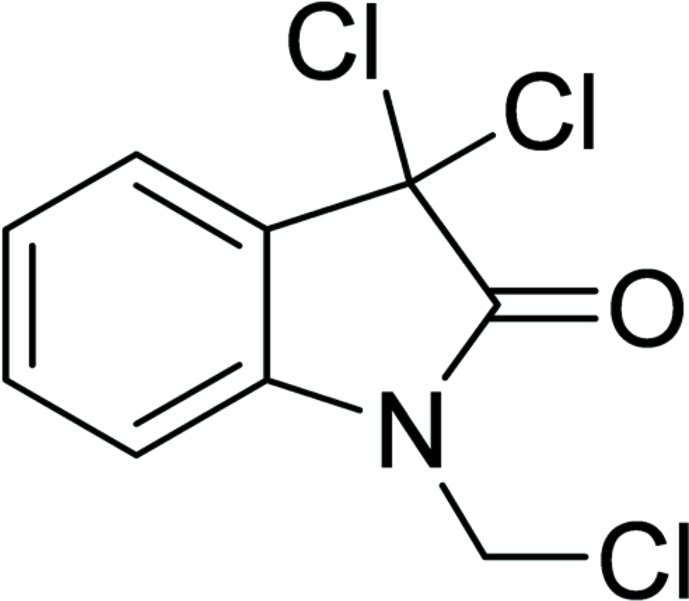


## Experimental

### 

#### Crystal data

C_9_H_6_Cl_3_NO
*M* *_r_* = 250.50
Monoclinic, 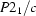

*a* = 8.6102 (1) Å
*b* = 14.5573 (2) Å
*c* = 8.2461 (1) Å
β = 93.381 (1)°
*V* = 1031.78 (2) Å^3^
*Z* = 4
Mo *K*α radiation
μ = 0.85 mm^−1^
*T* = 296 K
0.16 × 0.12 × 0.10 mm

#### Data collection

Bruker APEXII CCD area-detector diffractometer
13146 measured reflections
2450 independent reflections
2156 reflections with *I* > 2σ(*I*)
*R*_int_ = 0.016

#### Refinement

*R*[*F* ^2^ > 2σ(*F* ^2^)] = 0.035
*wR*(*F* ^2^) = 0.094
*S* = 1.06
2450 reflections
127 parameters
H-atom parameters constrained
Δρ_max_ = 0.41 e Å^−3^
Δρ_min_ = −0.49 e Å^−3^

### 

Data collection: *APEX2* (Bruker, 2007 ▶); cell refinement: *APEX2* and *SAINT* (Bruker, 2007 ▶); data reduction: *SAINT*; program(s) used to solve structure: *SHELXS97* (Sheldrick, 2008 ▶); program(s) used to refine structure: *SHELXL97* (Sheldrick, 2008 ▶); molecular graphics: *SHELXTL* (Sheldrick, 2008 ▶); software used to prepare material for publication: *SHELXTL* and *PLATON* (Spek, 2009 ▶).

## Supplementary Material

Crystal structure: contains datablocks I, global. DOI: 10.1107/S1600536810040717/kj2158sup1.cif

Structure factors: contains datablocks I. DOI: 10.1107/S1600536810040717/kj2158Isup2.hkl

Additional supplementary materials:  crystallographic information; 3D view; checkCIF report

## Figures and Tables

**Table 1:** Hydrogen-bond geometry (Å, °)

*D*—H⋯*A*	*D*—H	H⋯*A*	*D*⋯*A*	*D*—H⋯*A*
C4—H4⋯O1^i^	0.93	2.57	3.173 (2)	123

Symmetry code: (i) 
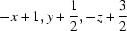
.

**Fig. 1.**
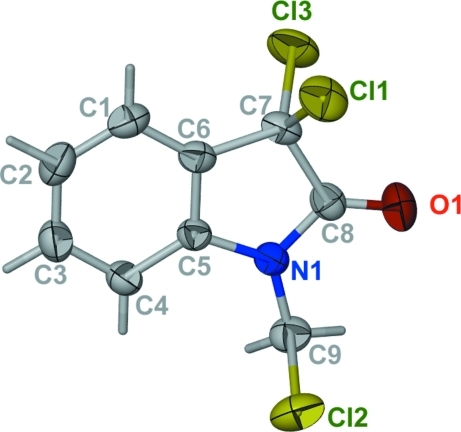
The title molecule with the atomic numbering scheme. The displacement ellipsoids are shown at the 50% probability level.

**Fig. 2.**
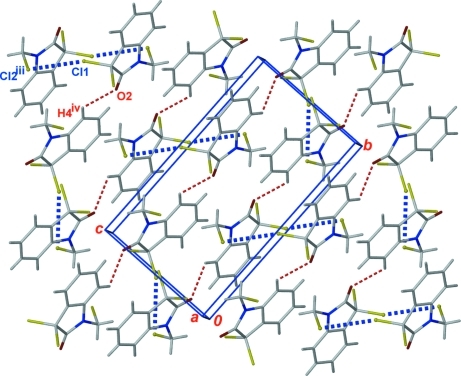
Cl···Cl and C-H···O=C interactions in the crystalline structure of the title compound. The blue dashed lines indicate Cl···Cl interaction, while the red dashed lines represent C—H···O=C interactions. All the π–π stacking interactions are omitted for clarity. Symmetry codes: iii-x + 1, -y + 1, -z + 2; iv-x + 1, y - 1/2, -z + 3/2.

## supplementary crystallographic information

### Comment

Höhme and Schwartz reported that the reaction of 1-(hydroxymethyl) indoline-2,3-dione with SOCl_2 _gave 1-(chloromethyl) indoline-2,3-dione (Höhme & Schwartz, 1973), whereas the reaction in the presence of a small amount of pyridine gave the title compound (Höhme & Schwartz, 1974). However, our experimental results showed that the title compound could also be obtained from the reaction in the absence of pyridine. Here, we report the structure of the title compound.

X-ray crystal analysis shows that the pyrrole ring almost lies within the plane of the benzene ring, while the torsion angle Cl2—C9—N1—C8 equals 98.78 (17)°, as shown in Fig.1. Two molecules arrange in a face to face mode and thus interconnect through intermolecular Cl1···Cl2(-*x* + 1, -*y* + 1, -*z* + 2) interactions (Cl···Cl=3.564 (5) Å) (Reddy *et al.*, 2006), forming a dimer. Each dimeric unit peripherically links to four neihgbouring ones through intermolecular C4—H4···O1=C8 interactions (Table 1), generating a two-dimensional network. π–π interactions (Table 2) between the approximate parallel benzene and/or parrole rings cooperate with those weak interactions to consolidate the supramolecular structure, as shown in Fig. 2.

### Experimental

A mixture of indoline-2,3-dione (3.0 g, 0.02 mol) and formalin (5 ml) in 30 ml of water was refluxed for 1 h. After that, the reaction mixture was stirred at room temperature overnight. The resulting precipitate, 1-(hydroxymethyl)indoline-2,3-dione, was separated by filtration and purified by recrystallization from ethanol, which was heated with SOCl_2 _(25 ml) under reflux for 3.5 h. The reaction mixture was distilled in vacuum to remove excess SOCl_2 _and the residue was purified by column chromatography on silica gel using dichloromethane/methanol=98:2, *v*/*v*, as an eluent (*R_f_*=0.33, dichloromethane/methanol=98:2, *v*/*v*; m.p. 141–143°C; yield 50.5% in two steps). The light yellow crystals of the title compound were obtained by slow evaporation from the solution of dichloromethane methanol 98:2 (*v*/*v*) at room temperature.

### Refinement

All the H atoms were discernible in the difference electron density maps. Nevertheless, the hydrogen atoms were placed into idealized positions and allowed to ride on the carrier atoms, with C—H=0.93 Å for aryl H atoms and *U_iso_*(H)=1.2*U_eq_*(C).

### Crystal data

**Table:**

C_9_H_6_Cl_3_NO	*F*(000) = 504
*M**_r_* = 250.50	*D*_x_ = 1.613 Mg m^−^^3^*D*_m_ = 1.613 Mg m^−^^3^*D*_m_ measured by not measured
Monoclinic, *P*2_1_/*c*	Mo *K*α radiation, λ = 0.71073 Å
*a* = 8.6102 (1) Å	Cell parameters from 7007 reflections
*b* = 14.5573 (2) Å	θ = 2.5–27.8°
*c* = 8.2461 (1) Å	µ = 0.85 mm^−^^1^
β = 93.381 (1)°	*T* = 296 K
*V* = 1031.78 (2) Å^3^	Block, yellow
*Z* = 4	0.16 × 0.12 × 0.10 mm

### Data collection

**Table:**

Bruker APEXII CCD area-detector diffractometer	2156 reflections with *I* > 2σ(*I*)
Radiation source: fine-focus sealed tube	*R*_int_ = 0.016
graphite	θ_max_ = 27.9°, θ_min_ = 2.4°
phi and ω scans	*h* = −11→11
13146 measured reflections	*k* = −19→19
2450 independent reflections	*l* = −10→10

### Refinement

**Table:**

Refinement on *F*^2^	Primary atom site location: structure-invariant direct methods
Least-squares matrix: full	Secondary atom site location: difference Fourier map
*R*[*F*^2^ > 2σ(*F*^2^)] = 0.035	Hydrogen site location: inferred from neighbouring sites
*wR*(*F*^2^) = 0.094	H-atom parameters constrained
*S* = 1.06	*w* = 1/[σ^2^(*F*_o_^2^) + (0.0433*P*)^2^ + 0.4377*P*] where *P* = (*F*_o_^2^ + 2*F*_c_^2^)/3
2450 reflections	(Δ/σ)_max_ < 0.001
127 parameters	Δρ_max_ = 0.41 e Å^−^^3^
0 restraints	Δρ_min_ = −0.49 e Å^−^^3^

### Special details

**Table:**

Geometry. All e.s.d.'s (except the e.s.d. in the dihedral angle between two l.s. planes) are estimated using the full covariance matrix. The cell e.s.d.'s are taken into account individually in the estimation of e.s.d.'s in distances, angles and torsion angles; correlations between e.s.d.'s in cell parameters are only used when they are defined by crystal symmetry. An approximate (isotropic) treatment of cell e.s.d.'s is used for estimating e.s.d.'s involving l.s. planes.
Refinement. Refinement of *F*^2^ against ALL reflections. The weighted *R*-factor *wR* and goodness of fit *S* are based on *F*^2^, conventional *R*-factors *R* are based on *F*, with *F* set to zero for negative *F*^2^. The threshold expression of *F*^2^ > σ(*F*^2^) is used only for calculating *R*-factors(gt) *etc*. and is not relevant to the choice of reflections for refinement. *R*-factors based on *F*^2^ are statistically about twice as large as those based on *F*, and *R*- factors based on ALL data will be even larger.

### Fractional atomic coordinates and isotropic or equivalent isotropic displacement parameters (Å^2^)

**Table:**

	*x*	*y*	*z*	*U*_iso_*/*U*_eq_	
C9	0.4139 (2)	0.66723 (13)	0.5958 (2)	0.0448 (4)	
H9A	0.3747	0.6171	0.5275	0.054*	
H9B	0.4404	0.7176	0.5255	0.054*	
Cl1	0.72991 (7)	0.48322 (4)	0.99637 (6)	0.06304 (17)	
Cl3	0.87749 (7)	0.48989 (4)	0.69017 (7)	0.06607 (17)	
Cl2	0.26419 (5)	0.70402 (4)	0.72483 (7)	0.06448 (17)	
C1	0.9042 (2)	0.68812 (13)	0.9240 (2)	0.0478 (4)	
H1	0.9838	0.6541	0.9762	0.057*	
C2	0.9062 (2)	0.78348 (14)	0.9279 (3)	0.0533 (4)	
H2	0.9882	0.8138	0.9836	0.064*	
C3	0.7883 (2)	0.83366 (12)	0.8503 (2)	0.0497 (4)	
H3	0.7922	0.8975	0.8545	0.060*	
C4	0.66346 (19)	0.79124 (11)	0.7659 (2)	0.0415 (4)	
H4	0.5837	0.8252	0.7139	0.050*	
C5	0.66323 (17)	0.69655 (10)	0.76292 (19)	0.0345 (3)	
C6	0.78112 (18)	0.64512 (11)	0.84075 (19)	0.0373 (3)	
C7	0.74369 (19)	0.54609 (11)	0.8146 (2)	0.0410 (3)	
C8	0.58313 (19)	0.54734 (11)	0.7189 (2)	0.0402 (3)	
N1	0.54994 (15)	0.63781 (9)	0.68728 (17)	0.0385 (3)	
O1	0.50408 (18)	0.48209 (9)	0.68047 (17)	0.0562 (3)	

### Atomic displacement parameters (Å^2^)

**Table:**

	*U*^11^	*U*^22^	*U*^33^	*U*^12^	*U*^13^	*U*^23^
C9	0.0434 (8)	0.0522 (9)	0.0379 (8)	0.0048 (7)	−0.0044 (7)	−0.0021 (7)
Cl1	0.0768 (3)	0.0574 (3)	0.0547 (3)	0.0061 (2)	0.0024 (2)	0.0214 (2)
Cl3	0.0658 (3)	0.0575 (3)	0.0769 (4)	0.0237 (2)	0.0207 (3)	−0.0084 (2)
Cl2	0.0411 (2)	0.0907 (4)	0.0615 (3)	0.0133 (2)	0.0021 (2)	−0.0031 (3)
C1	0.0344 (8)	0.0575 (10)	0.0512 (10)	0.0034 (7)	−0.0008 (7)	0.0031 (8)
C2	0.0421 (9)	0.0578 (11)	0.0595 (11)	−0.0122 (8)	−0.0006 (8)	−0.0053 (9)
C3	0.0509 (10)	0.0381 (8)	0.0609 (11)	−0.0072 (7)	0.0093 (8)	−0.0033 (8)
C4	0.0415 (8)	0.0350 (7)	0.0482 (9)	0.0034 (6)	0.0053 (7)	0.0020 (6)
C5	0.0332 (7)	0.0345 (7)	0.0361 (7)	0.0023 (5)	0.0045 (6)	−0.0008 (6)
C6	0.0358 (7)	0.0380 (8)	0.0386 (8)	0.0064 (6)	0.0062 (6)	0.0018 (6)
C7	0.0452 (8)	0.0369 (8)	0.0416 (8)	0.0106 (6)	0.0072 (7)	0.0039 (6)
C8	0.0473 (8)	0.0355 (7)	0.0383 (8)	0.0015 (6)	0.0075 (7)	−0.0014 (6)
N1	0.0379 (6)	0.0344 (6)	0.0428 (7)	0.0032 (5)	−0.0013 (5)	−0.0021 (5)
O1	0.0701 (9)	0.0399 (6)	0.0583 (8)	−0.0114 (6)	0.0012 (7)	−0.0040 (6)

### Geometric parameters (Å, °)

**Table:**

C9—N1	1.422 (2)	C3—C4	1.390 (2)
C9—Cl2	1.8009 (18)	C3—H3	0.9300
C9—H9A	0.9700	C4—C5	1.379 (2)
C9—H9B	0.9700	C4—H4	0.9300
Cl1—C7	1.7664 (17)	C5—C6	1.388 (2)
Cl3—C7	1.7856 (16)	C5—N1	1.414 (2)
C1—C6	1.378 (2)	C6—C7	1.490 (2)
C1—C2	1.389 (3)	C7—C8	1.551 (2)
C1—H1	0.9300	C8—O1	1.200 (2)
C2—C3	1.378 (3)	C8—N1	1.369 (2)
C2—H2	0.9300		
			
N1—C9—Cl2	111.84 (12)	C4—C5—C6	122.06 (15)
N1—C9—H9A	109.2	C4—C5—N1	127.79 (14)
Cl2—C9—H9A	109.2	C6—C5—N1	110.15 (13)
N1—C9—H9B	109.2	C1—C6—C5	120.33 (15)
Cl2—C9—H9B	109.2	C1—C6—C7	131.67 (15)
H9A—C9—H9B	107.9	C5—C6—C7	107.99 (14)
C6—C1—C2	118.30 (16)	C6—C7—C8	103.95 (12)
C6—C1—H1	120.9	C6—C7—Cl1	113.78 (12)
C2—C1—H1	120.9	C8—C7—Cl1	109.59 (12)
C3—C2—C1	120.73 (17)	C6—C7—Cl3	112.65 (12)
C3—C2—H2	119.6	C8—C7—Cl3	107.40 (11)
C1—C2—H2	119.6	Cl1—C7—Cl3	109.15 (8)
C2—C3—C4	121.61 (16)	O1—C8—N1	127.02 (16)
C2—C3—H3	119.2	O1—C8—C7	126.84 (15)
C4—C3—H3	119.2	N1—C8—C7	106.13 (13)
C5—C4—C3	116.96 (16)	C8—N1—C5	111.52 (13)
C5—C4—H4	121.5	C8—N1—C9	123.16 (14)
C3—C4—H4	121.5	C5—N1—C9	125.26 (13)
			
C6—C1—C2—C3	−0.1 (3)	C6—C7—C8—O1	−175.08 (17)
C1—C2—C3—C4	0.1 (3)	Cl1—C7—C8—O1	−53.1 (2)
C2—C3—C4—C5	−0.2 (3)	Cl3—C7—C8—O1	65.3 (2)
C3—C4—C5—C6	0.3 (2)	C6—C7—C8—N1	4.91 (17)
C3—C4—C5—N1	179.42 (16)	Cl1—C7—C8—N1	126.87 (12)
C2—C1—C6—C5	0.2 (3)	Cl3—C7—C8—N1	−114.67 (12)
C2—C1—C6—C7	−179.47 (18)	O1—C8—N1—C5	174.89 (17)
C4—C5—C6—C1	−0.3 (2)	C7—C8—N1—C5	−5.10 (18)
N1—C5—C6—C1	−179.56 (15)	O1—C8—N1—C9	−2.4 (3)
C4—C5—C6—C7	179.42 (15)	C7—C8—N1—C9	177.62 (14)
N1—C5—C6—C7	0.16 (18)	C4—C5—N1—C8	−175.89 (16)
C1—C6—C7—C8	176.63 (17)	C6—C5—N1—C8	3.31 (19)
C5—C6—C7—C8	−3.04 (17)	C4—C5—N1—C9	1.3 (3)
C1—C6—C7—Cl1	57.5 (2)	C6—C5—N1—C9	−179.48 (15)
C5—C6—C7—Cl1	−122.18 (13)	Cl2—C9—N1—C8	98.78 (17)
C1—C6—C7—Cl3	−67.4 (2)	Cl2—C9—N1—C5	−78.12 (19)
C5—C6—C7—Cl3	112.91 (13)		

### Hydrogen-bond geometry (Å, °)

**Table:**

*D*—H···*A*	*D*—H	H···*A*	*D*···*A*	*D*—H···*A*
C4—H4···O1^i^	0.93	2.57	3.173 (2)	123
C9—H9A···O1	0.97	2.56	2.879 (2)	100
C9—H9A···O1^ii^	0.97	2.52	3.256 (2)	133

Symmetry codes: (i) −*x*+1, *y*+1/2, −*z*+3/2; (ii) −*x*+1, −*y*+1, −*z*+1.

### Table 2 π–π interactions

**Table:**

Cg(A)	Cg(B)	Cg(A)···Cg(B) (Å)	sym. code Cg(B)
Cg1	Cg1	4.134 (7)	x, -y +3/2, z-1/2
Cg1	Cg1	4.134 (6)	x, -y +3/2, z+1/2
Cg1	Cg2	4.238 (7)	x, -y +3/2, z-1/2

^*^ Cg1, Cg2 are the centroids of C1-C2-C3-C4-C5-C6 (benzene) and C5-C6-C7-C8-N1 (pyrrole),respectively.
